# Metro-Peritonitis Again

**Published:** 1871-07

**Authors:** Theodore Griffin

**Affiliations:** 582 State Street


### METRO-PERITONITIS AGAIN.

Prof. Davis,—I have recently had my attention called to a letter which appeared over the signature "Viatus," in a late number of a medical journal published in this city. Said letter professes to review the case of metro-peritonitis which I reported in the Examiner, but it is of such a character as to be almost unworthy of any notice. In it the writer shows himself more familiar with the peculiar phraseology of the prize ring than with the principles of medical diagnosis.

He declares that I made a false diagnosis, that the disease was not metro-peritonitis. I think it well to consider briefly the merit of that declaration. He attempts to make a strong point upon the character of the pulse as reported by me. In my report the character of the pulse as to volume was alluded to but once, and that was during the prodromic period, before the symptoms of metro-peritonitis were fully initiated. The pulse, in severe peritoneal inflammation, is generally quick, feeble, and jerking, though it may be hard and full. After the full development of peritoneal inflammation the pulse ranges from 110 to 150 per minute, *when uninfluenced by medication.* Is the physician who controls the circulation by medication, so as to prevent it reaching 120 or 150 beats per minute, to be told in consequence that he has made a false diagnosis? In the case reported, all the grave and characteristic symptoms of metro-peritonitis were present in a marked degree. I do not consider it necessary to enumerate them here. The sudden distension of the abdomen, which sometimes occurs within a few hours after confinement, cannot be mistaken for metro-peritonitis. This distension is unaccompanied by the pain, fever, or other characteristic symptoms of metro-peritonitis.

I wish to say a word with reference to the duration of the case reported by me. It seems unusually short. It is so, the period of active inflammatory action and convalesence having occupied not more than ten or twelve days. I am satisfied that the inflammatory action was abruptly cut short in this instance by the opium. As soon as the opium impression was manifested the pulse was held at 110, and thereafter gradually became slower. Such, also, is the experience of Alonzo Clark; the pulse falling from 120 to 90 and 68 per minute, the disease often running a remarkably short course. If evidence is worth anything, it seems to me that it points conclusively to the value of the opium treatment of this disease. The fact that opium poisoning may occur, unless great caution be exercised in its use, will not deter the physician from a fair and thorough trial of the remedy.

I apologise for obtruding this subject again upon the attention of the readers of the EXAMINER. I assure you, however, that this closes what I have to say upon the subject. Permit me to say, in closing, to those individuals who have been so busily collecting items in regard to my case, that had they called upon me they might have saved a great amount of time and trouble in interviewing the physicians and druggists in my vicinity. I could have given them more information on the subject in twenty minutes than they could have received from my neighbors in an hour. Yours truly,

THEODORE GRIFFIN, M.D.

582 State Street.

